# A Quality Improvement Initiative to Improve Patient Safety Event Reporting by Residents

**DOI:** 10.1097/pq9.0000000000000519

**Published:** 2022-01-21

**Authors:** Daniel Herchline, Christina Rojas, Amit A. Shah, Victoria Fairchild, Sanjiv Mehta, Jessica Hart

**Affiliations:** From the Department of Pediatrics, Children’s Hospital of Philadelphia, Philadelphia, Pa.

## Abstract

**Introduction:**

Residents play a key role in patient care at academic medical centers and have unique insights into safety improvement opportunities. At our institution, <1% of safety events were reported by resident trainees. The primary objective of this quality improvement (QI) initiative was to increase the monthly incidence of event reporting by pediatric residents by 20% from baseline within 12 months.

**Methods:**

A QI team used the model for improvement to identify barriers to submitting safety event reports. The team used multiple intervention cycles to increase knowledge and promote engagement in event reporting. Interventions included educational tip sheets, a hospital-wide Morbidity and Mortality (M&M) conference, peer recognition and acknowledgment by senior leadership for report submission, and an interactive reporting activity. The outcome measure was monthly number of reports filed by residents. The process measure was the number of unique residents submitting a report each month. Time to complete a report was a balancing measure.

**Results:**

The number of reports placed by residents increased significantly, with a centerline shift from 15 to 29 reports per month (statistical process control chart—Fig. 3). The number of unique residents submitting reports increased from 10 to 22 per month. The time to complete a report was unchanged.

**Conclusions:**

Engaging residents in patient safety initiatives through education, experiential learning, and recognition can increase safety event reporting by residents. Future planned interventions include enhancing safety event reporting technology, developing patient safety faculty and resident champions, and increasing transparency regarding outcomes of safety event reports.

## Abstract 2

Supplemental Digital Content is available in the text.

## INTRODUCTION

In recent years, the Association of American Medical Colleges and the Accreditation Council for Graduate Medical Education have identified engaging physician trainees in quality improvement (QI) and patient safety initiatives as a critical aspect of training.^1,2^ Several studies have highlighted the need to improve trainee competence in these fields, leading to augmented patient safety education.^3,4^ However, interventions have been heterogeneous, and the impact on patient outcomes is limited.^5^

Safety event reporting is a core tenet of institutional safety culture.^6^ However, physician and resident safety reports represent a small proportion of the total submitted safety event reports.^7,8^ This discrepancy represents a crucial missed opportunity, as graduate medical education trainees are uniquely positioned to contribute meaningfully to institutional patient safety culture given their perspective as front-line clinicians and their responsibility for a large volume of patient encounters.^9^ Previous studies examined this dearth of resident-driven safety event reporting, noting various barriers including lack of time, poor understanding of the safety event reporting process, and fear of retaliation.^10,11^ Several different interventions targeting these identified barriers have been trialed, including education, periodic trainee-led reviews of safety event reports, incentive programs, and provision of supplemental aids to guide trainees in report completion.^12–16^ However, there have been few efforts to date that utilize a multifaceted approach to improve trainees’ safety event reporting rates.^17,18^

### Rationale

At our institution, resident trainees accounted for less than 1% of the total safety event reports filed each month, approximately 15 per month. Additionally, residents were infrequently involved in root cause analyses of serious safety events. In January 2019, the institution developed the House Staff Quality and Patient Safety Council (HQSC), a trainee-run collaboration among house staff, the graduate medical education office, the Quality and Safety office, senior hospital leadership, and various interprofessional partners. One of the primary goals of this council was to promote the integration of house staff into the safety mission of the institution, mainly through involvement in institutional quality and safety initiatives. A team including several members of the HQSC conceptualized and implemented a QI initiative, using the Model for Improvement, to understand barriers to safety event reporting by trainees and to develop interventions that directly linked to the key drivers.^19^

### Specific Aim

The project’s SMART aim was to increase the monthly number of reports filed by residents by 20% from baseline by June 2020. The team selected 20% as an initial aim for the project given the multitude of barriers impacting safety event reporting by trainees, and the presumed challenges of changing safety culture within a large hospital system. The team employed a multifaceted approach to accomplish this aim, including implementing multiple educational interventions developed based on QI methodology and informed by feedback from key stakeholders. This initiative supports the overarching goal of improving trainee competence and integration into the institutional patient safety culture as an avenue to enhance patient outcomes.

## METHODS

### Context

Our institution is a 546-bed tertiary-care center located in an urban setting. The hospital has approximately 2.5 million combined inpatient and outpatient encounters each year. The residency program is typically a 3-year program and includes categorical pediatric residents, combined medicine-pediatric residents, and combined subspecialty pediatric residents. The size of the resident class is roughly equal from year to year. In this QI initiative, we included reporting by residents split equally among the three years of training occurring across three fiscal years. The institution uses an electronic safety event reporting system to capture internally and externally accessible (web-based) events and events via the electronic health record.

### Interventions

**Fig. 1.**
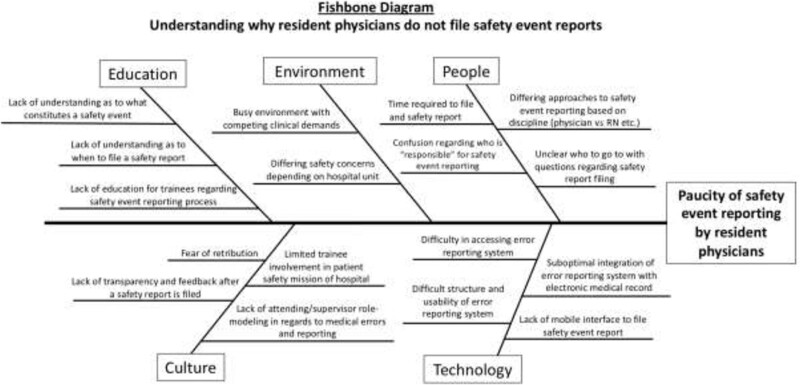
Fishbone diagram—Barriers to resident safety event reporting.

**Fig. 2.**
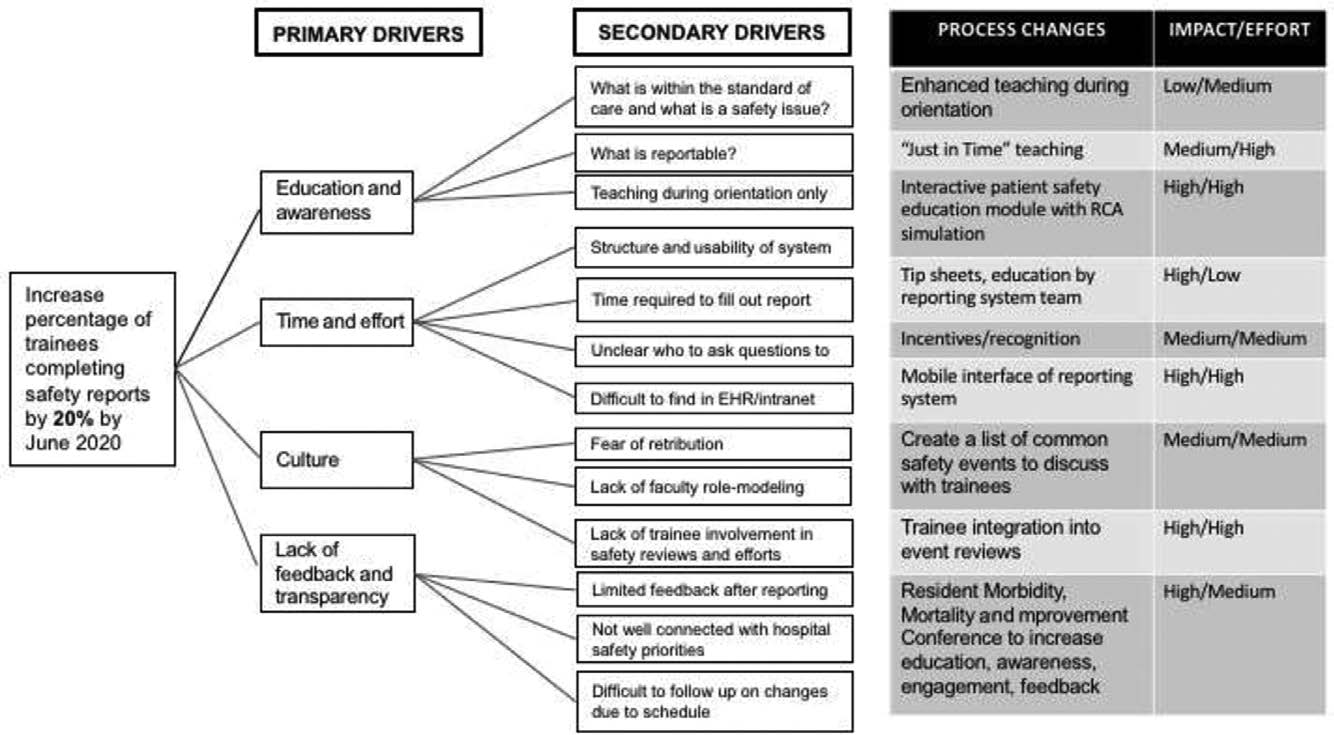
Driver diagram.

To better understand resident barriers to complete safety event reports, the HQSC team, including resident representatives covering all academic years, performed a root cause analysis using common improvement tools, including a fishbone diagram, a key driver diagram, a process map, and voice of the customer. They identified several contributing factors (Fig. 1) and four primary drivers: (1) education and awareness of the institution’s safety event reporting system; (2) time and effort associated with report submissions; (3) safety culture; and (4) lack of feedback and transparency surrounding reports (Fig. 2).

The team subsequently identified and engaged key stakeholders, including pediatric residency program leadership, the safety event reporting system team, and the institutional patient safety team. Together, these groups reviewed and discussed the root cause analysis results, partnering to develop collaborative, sustainable interventions. They chose to focus on the following key barriers to safety event reporting: culture, education, and awareness.

The team made an impact-effort matrix to select and prioritize possible interventions and focused on four key interventions (**see Appendix 1, Supplemental Digital Content 1,** http://links.lww.com/PQ9/A355). Each improvement cycle consisted of one intervention and targeted all residents simultaneously except cycle 4. Each cycle lasted approximately 1 month and was followed by an analysis period, in which the intervention was adopted, adapted, or abandoned. New interventions were developed based on outcome data and stakeholder feedback.

The first improvement cycle targeted educating residents on the process of safety event reporting. In collaboration with the safety event reporting system team, a series of informative tip sheets were developed and distributed to all residents via email and posted in resident workrooms. Three tip sheets were created and distributed biweekly. The next improvement cycle targeted culture, education, and awareness via institution-wide, trainee-run morbidity and mortality (M&M) conference focusing on psychological safety and the importance of reporting errors. The institutional M&M conference, titled “Being Vulnerable and Learning from Errors,” involved several high-profile hospital leaders, including the residency’s program director, sharing personal experiences with patient safety events. These speakers discussed the concept of psychological safety, in which individuals feel empowered and comfortable addressing challenging scenarios in the clinical care environment. This conference was broadcast to all institutional trainees and faculty. The success and positive trainee feedback related to this conference led to creating a bimonthly trainee-led pediatric resident morbidity, mortality, and improvement (MM&I) conference during which patient safety events are studied and discussed. In the third improvement cycle, culture and awareness were targeted by developing two separate recognition programs to highlight residents who had submitted safety event reports in the prior month. Residency program leadership led the first program via an acknowledgment of residents in program-wide emails. In the second program, a senior hospital leader contacted and recognized individual residents for their contribution to an institutional culture of safety. There were no financial or material incentives included in either of these programs. The final improvement cycle focused on improving education and awareness by engaging a subgroup of first-year residents in a novel, interactive “escape-room” experience designed to promote important patient safety principles, including safety event reporting and root cause analyses. This activity culminated with residents filing a hypothetical safety event report using the institution’s safety reporting system. Of note, the final improvement cycle was mandatory for all first-year residents, though it did not commence until halfway through the year. The other interventions were not required for residents. Approximately, 75% of all residents participate in daily conferences, which include the MM&I series.

### Study of the Interventions

The team collated safety event reports filed by residents with the assistance of the safety event reporting team. The team collected monthly data retrospectively for 1 year before the first study intervention (“preintervention” period: July 2018–July 2019), for 4 months during the interventions (August 2019–November 2019), and 9 months following the interventions (December 2019–August 2020), collectively the “postintervention” period. Finally, the HQSC analyzed all safety event reports filed by residents during the postintervention periods to identify potential themes warranting further investigation by institutional patient safety leadership, and categorized reports according to severity, including serious safety event, near miss events, event with harm, or event without harm.

### Measures

The primary outcome was the total number of safety event reports filed by residents each month. For process measures, the team tracked the number of unique residents filing safety event reports, both the total and number by year of training, as well as the number of residents who submitted reports within two months of completing the “escape room” session. We used the average time required by residents to complete a safety event report as a balancing measure because “time” was a known reporting barrier. Of note, this does not include the amount of time included for event follow-up.

### Data Analysis

The primary outcome measure—the total number of safety event reports filed by residents—was analyzed using a statistical process control chart (c-chart). A c-chart was also used to evaluate the process measure of unique residents filing safety event reports. Standard rules to determine special cause variation were employed.^20^

### Ethical Considerations

This project was undertaken as a QI initiative and does not constitute human subjects research. This project was adjudicated as QI work and was deemed exempt from IRB review. The article was written following the Standards for Quality Improvement Reporting Excellence 2.0 guidelines.^21^

## RESULTS

**Fig. 3.**
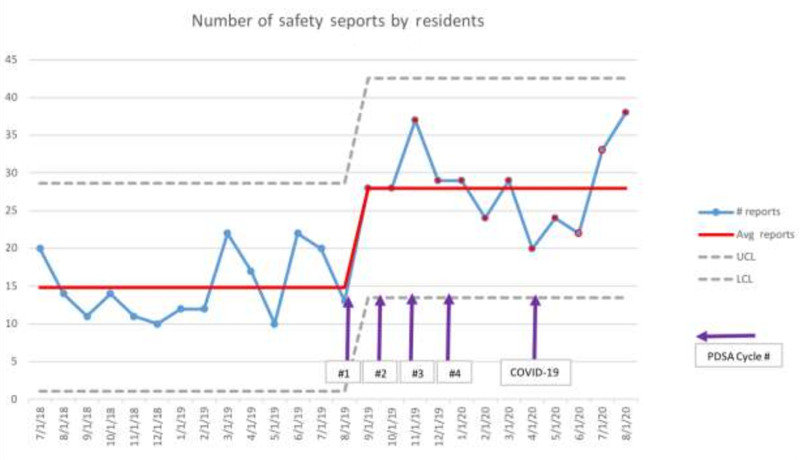
Statistical process control c-chart of the total number of safety event reports filed by residents per month. LCL, lower control limit; PDSA, plan, do, study, act; UCL, upper control limit.

During the preintervention period, the average number of patient safety event reports filed by residents was 15 per month, ranging from 10 to 22. The residents’ number of safety event reports improved after the first improvement cycle and reached special cause variation in months 3–5 (Fig. 3). After the 4 months of interventions, there was a significant improvement, and a centerline shift to 29 reports per month was established after eight consecutive months of improvement. The increase in safety reports filed by residents was sustained for 9 months following the last intervention. Notably, there was a decrease in the number of reports and individual residents reporting in April 2020, when residents were briefly taken out of clinical care and hospital census was dramatically lower due to COVID-19, but this improved in subsequent months as residents returned to service. There were no instances of duplicate resident reports. The number of safety event reports by other staff was not tracked.

**Fig. 4.**
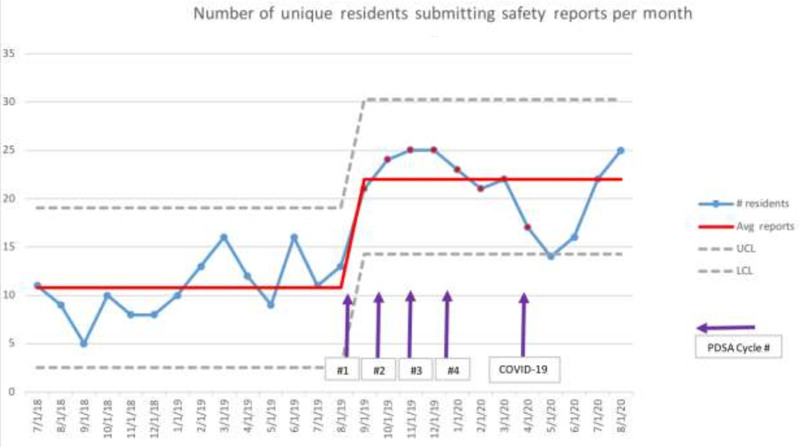
Statistical process control c-chart of the total number of unique residents filing safety event reports per month. LCL, lower control limit; PDSA, plan, do, study, act; UCL, upper control limit.

Process measures also demonstrated improvement. The number of unique residents submitting reports rose from an average of ten residents per month in the preintervention period to a centerline shift of 22 residents per month postinterventions (Fig. 4). The initiative was associated with a 49% increase in the total number of reports and a 34% increase in the total number of residents who submitted reports (Table 1). Regarding the number of reports, the first- and second-year residents showed a significant increase while the third year remained the same. Twenty-first-year residents participated in the patient safety “escape room” simulation. Four (20%) residents had submitted a report prior, and 15 (75%) submitted a report in the 2 months following the training activity.

**Table 1.** Resident Safety Event Reporting by Year in Residency Training

	No. Reports		No. Unique Residents Reporting	
	Preintervention	Postintervention	Preintervention	Postintervention
PGY1 FY19 N = 57 FY20 N = 58 FY21 N = 57	38	50	22	31
PGY2 FY19 N = 50 FY20 N = 52 FY21 N = 58	81	168	26	44
PGY3 FY19 N = 52 FY20 N = 50 FY21 N = 51	72	67	28	27
Total FY19 N = 159 FY20 N = 160 FY21 N = 166	191	285	76	102
% increase	49 (*P* < 0.01)		34 (*P* = 0.3)	

Preintervention and postintervention periods are 12 and 13 months in length, respectively.

FY, fiscal year; FY19, July 2018–June 2019; FY20, July 2019–June 2020; FY21, July 2020–June 2021; PGY1, first year; PGY2, second year; PGY3/4, third year and fourth year.

Finally, balancing metric data demonstrated that the average time required for residents to complete a safety event report remained unchanged at 8 minutes for both the preintervention and postintervention periods.

Each of the interventions was adopted with the exception of the M&M conference, which was adapted to become a monthly recurring event targeted toward residents (Table 2).

**Table 2.** Improvement Cycles and Outcomes of Interventions

Timeline	Improvement Cycle	Outcome
August–September 2019	#1: Tip sheets on using the reporting system to improve understanding and comfort	Adopted and spread: tip sheets were also distributed to faculty, and an instructional video was created for intern orientation in June 2020
October 2019	#2: Trainee-led, hospital-wide M&M conference that included both physician and nursing leadership on psychological safety and important of reporting errors	Adapted: residents started their own morbidity, mortality, and improvement conference to discuss events
November 2019	#3: Sustainable recognition system of residents by both a senior hospital leader and residency program leadership	Adopted
December 2019	#4: New interactive safety education module using gamification for interns	Adopted and spread: In FY21, all new interns will complete this module

## DISCUSSION

Using a multifaceted approach to address safety event reporting in trainees, the team increased the number of reports filed by residents within the institution. Additionally, the number of unique residents filing reports increased during the study period. This process measure was used to account for early adopters and resident champions who filled out multiple safety event reports each month, allowing us to assess our interventions’ breadth and depth of impact. Although impacted for several months by lower volumes due to COVID, the level of reporting was generally sustained over the subsequent 9 months. The initial root cause analysis revealed similar themes to those documented in the current literature.^10,11^ Notably, resident stakeholders highlighted several key concerns regarding patient safety culture. Residents noted that fear of retribution, lack of role-modeling by supervising physicians, and limited trainee involvement in the patient safety mission of the hospital all contribute to a lack of safety event reporting.^22,23^ Given these concerns, a central focus is developing interventions to improve resident engagement and understanding of safety culture. The institutional M&M conference, during which several high-profile hospital leaders shared their personal experiences with patient safety events and errors, helped strengthen the concept of psychological safety and the value of admitting mistakes to improve patient safety. Ultimately, the session provided an opportunity for residents to witness positive role-modeling by a well-respected, interprofessional group of leaders and hear from a trusted source that retribution does not occur following patient safety events.

Following this larger institutional session, the team partnered with resident stakeholders and residency program leadership to develop a monthly resident-led MM&I conference to create a more sustainable avenue for immersing residents into safety event analysis. This change added flexibility for scheduling when compared with the institutional session. Similar conferences have yielded positive results with respect to event reporting in trainees and residents have noted benefits in these MM&I sessions that provide further exposure to patient safety initiatives within the institution.^14,24^ Furthermore, they have provided a protected space for residents to discuss medical errors, fostering a psychologically safe work environment that promotes safety event reporting.^23^

The root cause analysis demonstrated that residents lack education regarding safety event reporting, both in logistics and importance. Although necessary, education alone is often not a high-impact intervention in QI initiatives.^25^ However, existing literature demonstrates that education can be useful in improving trainee safety event reporting. In this initiative, the use of tip sheets increased reporting.^4,17,18^ In the final intervention, the team sought to enhance the impact of education by pairing traditional educational tools with an innovative and novel pedagogy that embraces experiential learning.^26^ Gamification, the use of game design elements in a nongaming context to improve academic performance, and can improve trainee knowledge of patient safety concepts.^27,28^ Thus, the team developed and facilitated an “escape room” activity in which residents were introduced to a patient scenario in a simulation environment and, as a group, identified situational patient safety issues. This experience provided residents with an opportunity to learn core safety principles and allowed them to garner practical experience filling out safety event reports. Although this intervention occurred after special cause variation was already achieved and was only rolled out to a select group of first-year residents during this project, the team believed this was an important step to create sustainable education and engagement. The session is now part of the curriculum for all incoming first-year pediatric residents and has met with positive feedback.

Given prior successes in using incentive programs to incur positive trainee engagement in QI and patient safety initiatives, we opted to employ recognition as a possible way to motivate residents to complete safety event reports.^16,29^ Although there were no direct financial or material incentives for submitting reports, the team wanted to find ways to show residents appreciation for their efforts to improve patient safety. Although we did consider using financial incentives, we believed that it was reasonable to start with recognition given reported successes in prior studies.^30,31^ Notably, the recognition system run by residency program leadership was also created with the goal of increasing resident awareness and culture, as the list of residents who had filed safety reports was sent to the entire residency program.

Looking at a comparison by year of residency produced an interesting finding. Regarding the number of reports, the first- and second-year residents showed an increase while the third year residents showed no change. Much of the reporting increase was primarily due to the large number of second-year residents submitted reports. Although only speculation, it is possible that second-year residents have more understanding of the health care system and are in positions of increased autonomy at this point in their training, making them more likely to report systems-related events. Comparatively, the first-year residents may be acclimating to their new positions while the third-year residents may not have as much bandwidth to engage in organizational change, particularly if they are transitioning to different institutions following completion of training.

In analyzing the interventions, it is difficult to ascertain which had the most impact. Although the greatest increase in reports filed occurred after the initial intervention, we cannot conclude that this was the most successful intervention as the Hawthorne effect (resident awareness that safety event reporting was being tracked) likely contributed. Shifting the order of interventions may have produced a similar result. It is also possible that similar results could have been achieved with fewer cycles. However, in addressing multiple barriers, the team strove to increase the likelihood of positively impacting the overall safety culture of residents, as highlighted by a recent narrative review.^32^ Ultimately, the impact-effort matrix was used to guide the order and selection of interventions, to ensure sustainability and reach the greatest number of residents.

A major strength of this project was the multifaceted approach. In planning the interventions, the team consciously decided to address various barriers identified in the root cause analysis, rather than focusing on one. Lack of resident patient safety event reporting was a multifactorial issue, and thus required a similar approach. One of the most valuable aspects of this initiative is that each intervention continued once it was launched—creating a system in which new interventions were layered on older ones. The team believed this model had the effect of gradually and fundamentally changing the resident safety culture, leading to improved safety event reporting. The value of employing multifaceted approaches is similar to techniques used in rolling out care bundles for problems ranging from sepsis to central-line associated bacterial infections.^33,34^

There were several notable limitations to the initiative. The clustering of interventions made it challenging to determine the effect of individual intervention. Additionally, as previously mentioned, the escape-room experience was only available to first-year residents, highlighting the importance of the other interventions. Another key limitation is the lack of correlation with system changes made due to resident safety event reports. The initiative could also have been strengthened using a multicenter approach. Given that all institutions face unique challenges, some interventions may not generalize to other residency training programs without adaptation. Finally, this project did not focus on the quality or outcomes of event reports which was outside the scope of the original project.

## CONCLUDING SUMMARY

This multifaceted initiative, using QI tools and concepts, that engages residents in patient safety concepts through involvement in patient safety culture, experiential learning, and recognition can increase safety event reporting. Moving forward, the team plans to expand the pursuit of increasing resident engagement in institutional patient safety through various methods while collecting data on our measures to evaluate for sustained improvement. These include implementing an interdisciplinary system to improve transparency regarding safety event report outcomes, developing resident and faculty patient safety champions, and increasing resident involvement in systematic reviews of resident-reported safety events. It is critical to continue this work to further integrate patient safety into resident training and to ultimately provide a link to improved patient outcomes. We believe that this work can benefit other residency programs looking to improve trainee event reporting and, in turn, safety culture.

## ACKNOWLEDGMENTS

The authors thank the following people for their contributions to this project; the members of the HQSC for their dedicated time and efforts; the Residency Program leadership team for their support; Dr. Kathy Shaw, Dr. Jen Myers, and April Taylor, MS, MHA, CPPS, CPHQ, for their guidance and constructive recommendations on the manuscript; Kristin Mcnaughton, MHS, for her help with manuscript preparation and editing.

## DISCLOSURE

The authors have no financial interest to declare in relation to the content of this article.

## Supplementary Material


