# Supplementary material for: A Quality Improvement Initiative to Improve Patient Safety Event Reporting by Residents
Source: Pediatr Qual Saf. 2022 Jan 21;7(1):e519. doi: 10.1097/pq9.0000000000000519 (PMC8782116; doi:10.1097/pq9.0000000000000519)
Supplement: Supplementary file 1 [file pqs-7-e519-s001.pdf]

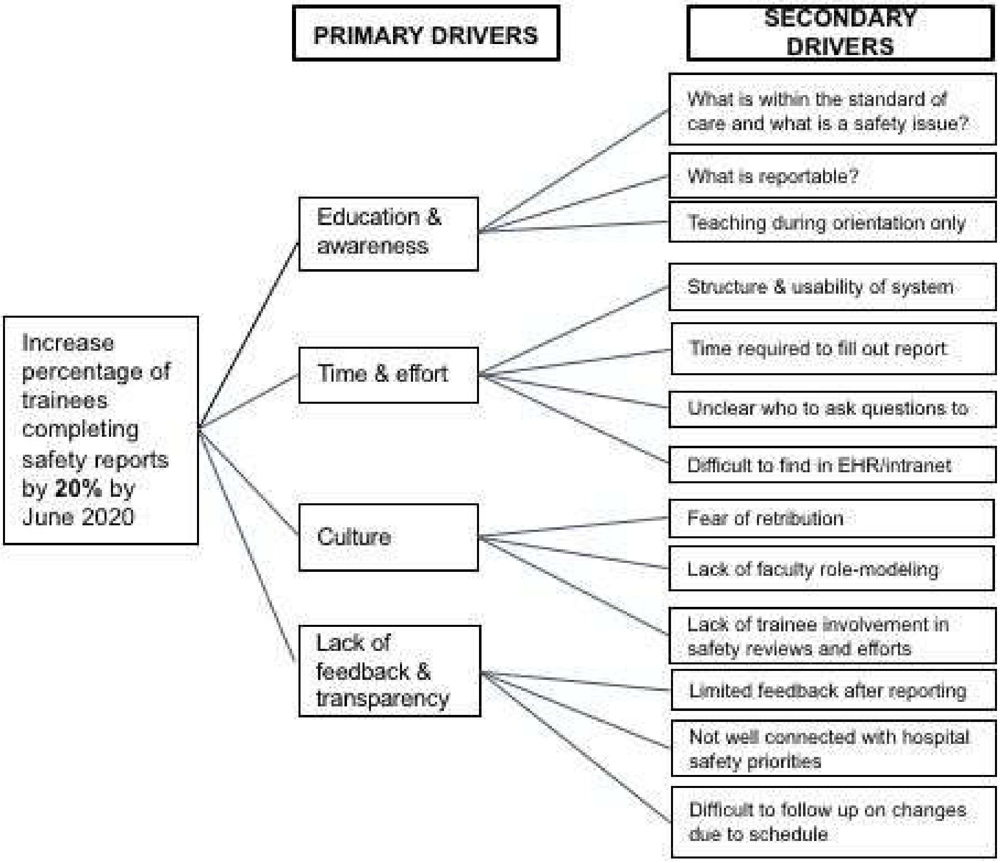

PROCESS CHANGES

Process Changes	Impact/Effort
Enhanced teaching during orientation	Low/Medium
"Just in Time" teaching	Medium/High
Interactive patient safety education module with RCA simulation	High/High
Tip sheets, education by reporting system team	High/Low
Incentives/recognition	Medium/Medium
Mobile interface of reporting system	High/High
Create a list of common safety events to discuss with trainees	Medium/Medium
Trainee integration into event reviews	High/High
Resident Morbidity, Mortality & Improvement Conference to increase education, awareness, engagement, feedback	High/Medium
